# Anti MDA-5 associated rapidly progressive interstitial lung disease complicated by viral pneumonia - a fatal outcome

**DOI:** 10.4322/acr.2024.511

**Published:** 2024-08-30

**Authors:** Saikat Mitra, Nithye Parvathy, Mandeep Garg, Shritik Devkota, Sandeep Bansal, Inderpaul Singh Sehgal, Kirti Gupta

**Affiliations:** 1 Postgraduate Institute of Medical Education and Research, Department of Histopathology, Chandigarh, India; 2 Postgraduate Institute of Medical Education and Research, Department of Pathology, Chandigarh, India; 3 Postgraduate Institute of Medical Education and Research, Department of Radiodiagnosis and imaging, Chandigarh, India; 4 Postgraduate Institute of Medical Education and Research, Department of Internal Medicine, Chandigarh, India; 5 Postgraduate Institute of Medical Education and Research, Department of Pulmonary Medicine, Chandigarh, India

**Keywords:** Dermatomyositis, Idiopathic Interstitial Pneumonias, Influenza A Virus, H1N1 Subtype, Lung Diseases, Interstitial Respiratory Distress Syndrome

## Abstract

Dermatomyositis is a heterogeneous systemic disease, with 7% to 10% of the individuals presenting the Anti MDA-5 antibody. This subset of patients has clinically amyotropic dermatomyositis, presenting with cutaneous ulcer and rapidly progressive interstitial lung disease. We report the case of a 22-year-old male with a six-month history of low-grade fever associated with myalgia, polyarthralgia, and marked weight loss. He had a history of shortness of breath and high-grade fever 15 days before admission. His clinical features and imaging workup were consistent with acute respiratory distress syndrome. A nasal swab was positive for H1N1 influenza virus infection. During the disease investigation, he succumbed after nine days of admission. The autopsy examination showed diffuse alveolar damage on a background of non-specific interstitial pattern of injury in the lungs. His postmortem muscle biopsy revealed subtle changes of inflammatory myopathy. The brain showed diffuse subarachnoid hemorrhage. Evaluation of postmortem serum sample revealed positivity for Anti MDA-5 and Ro-52 antibodies. This was a case of Anti MDA-5 and Ro-52 associated dermatomyositis with non-specific interstitial pneumonia pattern of lung injury complicated with H1N1 influenza pneumonia, leading to diffuse alveolar damage and subsequent respiratory failure and death. Serum Anti MDA-5 antibodies represent an important biomarker for diagnosing and predicting prognosis for patients with idiopathic inflammatory myopathies, especially clinically amyopathic dermatomyositis. Anti-Ro-52 has been reported in a wide variety of autoimmune diseases, particularly in myositis, scleroderma, and autoimmune liver diseases. Ro-52 autoantibodies are associated with interstitial lung disease (ILD), and their presence should encourage the clinician's curiosity to search for ILD.

## INTRODUCTION

Dermatomyositis (DM) is a heterogeneous disease with a broad spectrum of clinical manifestations ranging from classic DM, clinically amyopathic DM (CADM), and cancer-associated DM.^1^ Anti MDA-5 DM has a frequency ranging from 7 to 10% amongst adult DM.^2^ Anti MDA-5 DM is typically associated with cutaneous ulcerations, painful palmar papules, macules, and rapidly-progressive interstitial lung disease (RP-ILD), often with minimal muscle involvement.^3^ CADM has an aggressive and often fatal clinical course. CADM presentation is characterized by lung involvement, which may cause a delayed diagnosis due to the overlap with other causes of acute pneumonitis, such as infections, hypersensitivity pneumonitis, drug, and toxin-induced pneumonia. We discuss an autopsy case of Anti MDA-5 associated dermatomyositis presenting with RP-ILD, complicated by viral pneumonia. This case’s rarity and complex clinical and histological features make it unique for discussion.

## CASE REPORT

**Figure 1:**
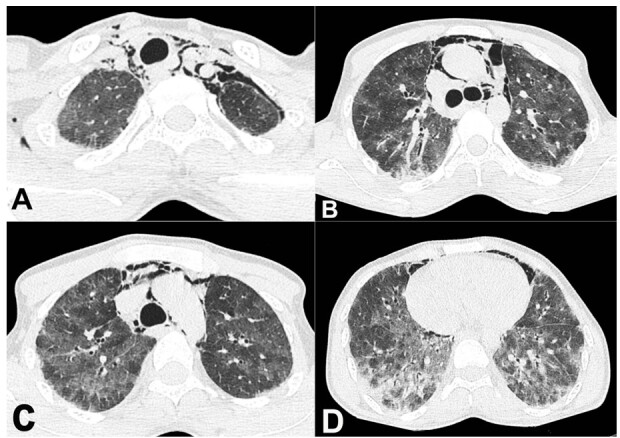
Thoracic HRCT - **A**, **B**, **C**, and **D** axial plane showing pneumomediastinum, subcutaneous emphysema, bilateral patchy areas of GGOs with interspersed septal thickening, and basal, subpleural, patchy consolidation. Also noted is a mild left-sided pneumothorax in **A**.

A 22-year-old male presented with a 15-day history of continuous high-grade fever associated with shortness of breath and retrosternal sharp, pricking chest pain over the last seven days. There was no orthopnea, paroxysmal nocturnal dyspnea, or wheezing. The patient had a history of low-grade on-and-off fever for the past six months. He also had polyarthralgia, symmetrically involving bilateral proximal interphalangeal, metacarpophalangeal, elbow, and knee joints and small joints of both feet, which were partly relieved with herbal medicine. He developed a generalized erythematous macular rash for six months. No redness or warmth of the joints was noted. No photosensitivity, sicca symptoms, dysphagia, alopecia, or Raynaud’s phenomenon was present. No cutaneous ulcers were noted. Three months after the onset of symptoms, the patient developed dry cough with a marked weight loss (15kg, almost 20% from initial weight) over six months. His medical history was non-contributory. On admission, he was tachypneic with room-air oxygen saturation of 80%. Chest auscultation revealed bilateral diffuse crepitation. No organomegaly, clubbing, cyanosis, icterus, or lymphadenopathy was detected. His muscle strength was symmetrically reduced (4/5); neurological and cardiovascular examinations were normal. The laboratory workup included hemogram, serum electrolytes, CPK, renal, liver, and thyroid function tests that were within normal limits. Serum C3 and C4 levels were normal, ANA, ANCA, rheumatoid factor, anti-CCP were negative. Tests for HIV, HBsAg, and Hepatitis C virus were negative. From the second day of admission, his total leukocyte count started rising, and on the day before demise, AST and ALT levels were markedly elevated [AST 1712 U/L (normal 5-42 u/l), ALT 1135 U/L (normal 5-40 u/l]. Thoracic high-resolution computed tomography (HRCT) showed multifocal areas of diffuse ground glass opacities (GGOs) in both lungs with subtle septal thickening in the background, and patchy consolidation in the lung bases. However, no traction bronchiectasis/bronchiolectasis or honeycombing was seen. In addition, pneumomediastinum, left-sided pneumothorax and subcutaneous emphysema extending into bilateral neck spaces were present (Figure 1A-D).

No joint deformities were identified on bilateral hands and feet X-ray. He was started on empirical antibiotics and high-flow oxygen through a nasal cannula, but was intubated soon after. Multiple blood culture tests did not yield any organism growth. Sputum and endotracheal aspirate were negative for bacteria, fungi, *Pneumocystis jirovecii,* and acid-fast bacilli. The endotracheal tube swab test for H1N1 virus was positive, and COVID-19 RT-PCR was negative. 2-D echocardiography was normal. He was started on oseltamivir drug on the fifth day of admission. His respiratory failure worsened; he had a single episode of tonic-clonic seizure and succumbed to his illness nine days after admission.

## AUTOPSY EXAMINATION

A complete autopsy was performed after an informed consent.

**Figure 2:**
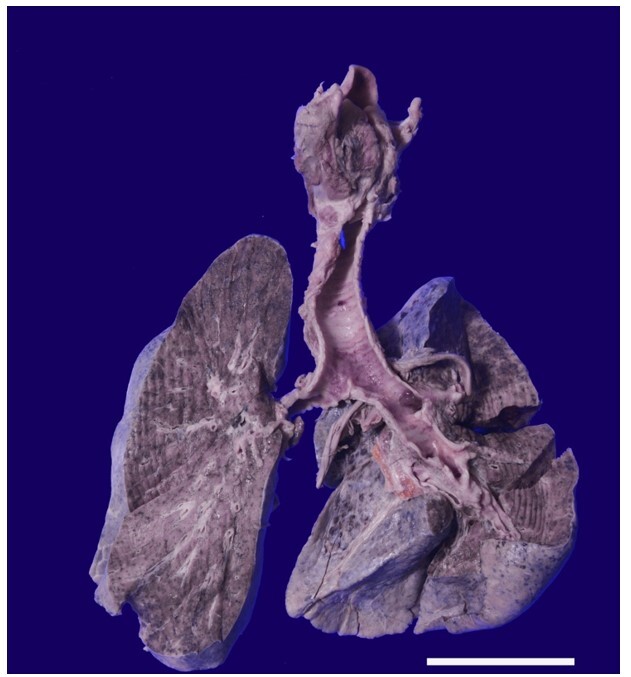
Cut surface of bilateral lungs and tracheobronchial tree shows no ulcer or aspirated material in the trachea. The cut surface of both lungs shows fibrotic patches. No cavity, space-occupying lesion, or hemorrhagic changes were identified on gross examination (Scale bar= 10 cm).

At the opening of the thoracic cavity, one liter of serous fluid drained from pleural cavities. Bilateral lungs were heavy (weight-900 g, normal range-500-700 g). Pleura was diffusely dull. The cut surface of both lungs was firm, non-crepitant, and slippery. No cavity or focal lesions were identified in both lungs (Figure 2).

**Figure 3:**
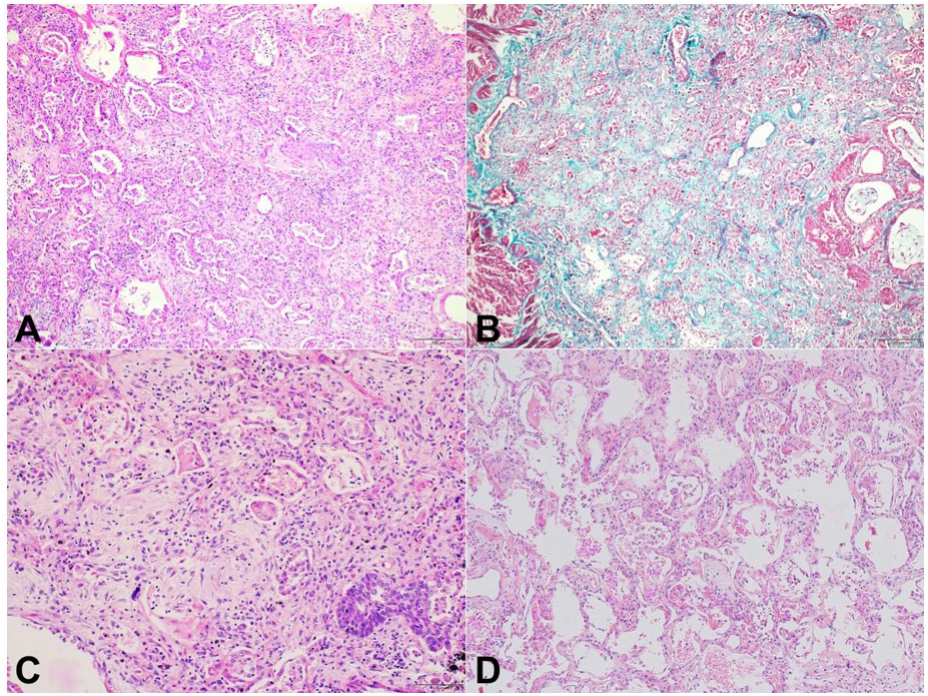
Photomicrographs of the lung. **A** and **B** – Microscopic examination of the lungs shows evidence of a proliferative phase of diffuse alveolar damage in the form of marked interstitial widening, loose myxoid fibroblastic proliferation, and intralveolar Masson’s body formation (**A**: H&E, 40x; **B**: MT, 40x); **C** – Diffuse type 2 pneumocyte hyperplasia and squamous metaplasia is identified (H&E, 400x); **D** – Sections from the grossly uninvolved lung parenchyma shows diffuse interstitial widening by fibrosis suggestive of non-specific interstitial pneumonia pattern of injury (H&E, 40x).

Pulmonary microscopic examination showed a proliferative phase of diffuse alveolar damage (DAD) (day 8-20) with marked interstitial widening, loose myxoid fibroblastic proliferation, and fibrosis with intra-alveolar Masson’s body formation (Figures 3A and 3B). Diffuse type-2 pneumocyte hyperplasia, focal squamous metaplasia, and scattered multinucleated pneumocytes were identified (Figure 3C). Sections from the relatively normal-looking lung parenchyma showed diffuse interstitial widening by fibrosis, suggestive of a non-specific interstitial pneumonia (NSIP) injury pattern (Figure 3D).

Areas of intra-alveolar hemorrhage were also noted. No vascular thrombi were identified. Ziehl-Neelsen, PAS, and Gram stains failed to show any micro-organisms. DNA and RNA were extracted from the fresh lung tissue and were subjected to viral PCR. COVID-19, H1N1, adenovirus, respiratory syncytial virus, metapneumovirus tests were negative. H1N1 immunohistochemistry was also performed on formalin-fixed paraffin-embedded tissue, which did not highlight viral inclusions.

**Figure 4:**
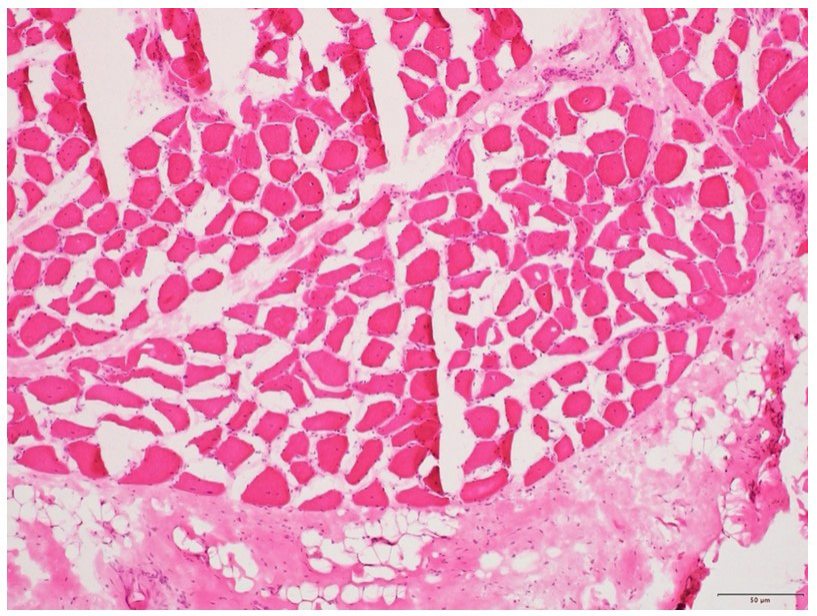
Photomicrograph of the muscle. Microscopic examination reveals no perifascicular atrophy, inflammatory infiltrate, or myonecrosis (H&E, 100x).

Muscle samples were obtained from the vastus lateralis, and deltoid and fresh muscle were snap-frozen. Microscopic examination revealed normal epi-, peri- and endomysium. There was no perifascicular atrophy, inflammatory infiltrate, or myonecrosis. Endomysial vessels appeared normal. However, mild fiber size variation, occasional nuclei internalization, and occasional myophagocytosis were noted (Figure 4).

Enzyme histochemistry was normal. Immunohistochemistry for membrane attack complex (MAC) was negative. No T-lymphocytes were detected on CD3, CD4, and CD8 immunohistochemistry. The skin showed no basal cell vacuolization, interface dermatitis, dermal mucin deposition, or vasculopathy changes. Microscopy from the liver revealed terminal shock-related ischemic changes in the form of centrizonal hepatocyte necrosis and sinusoidal congestion. The heart did not show any viral myocarditis or ischemic changes.

The kidneys, pancreas, spleen, thyroid, adrenals, testes, and gastrointestinal tracts were grossly and microscopically normal. Bone marrow showed trilineage hemopoiesis.

A post-mortem serum sample was collected during the autopsy and was tested for myositis-related antibodies. Immunoblot assay revealed strong 3+ positivity for Anti MDA-5 and Ro-52 antibodies and 1+ positivity for Anti-Mi2a antibodies.

Based on the clinical, histological, and serological findings, a final autopsy diagnosis of dermatomyositis (Anti MDA-5 associated), organizing phase of diffuse alveolar damage with underlying interstitial lung disease (NSIP pattern), and diffuse subarachnoid hemorrhage with tonsillar herniation was made.

## DISCUSSION

The initial reports of CADM with RP-ILD were published in Japan in 1990; however, Sato et al.^4^ reported a new antibody against CADM 140 antigen, which was subsequently demonstrated to be identical to MDA-5. MDA-5 is a Retinoic Acid Inducible Gene-1 (RIG-1)-like receptor coded by *IFIH1* (interferon-induced helicase C domain-containing protein 1) gene that recognizes double-stranded (ds) RNA of viruses and induces a type I interferon response through various intermediaries.^5^ The exact pathogenesis is unknown; however, a role of infections and environmental factors superimposed on genetic susceptibility has been proposed.^6^

Although the original descriptions of Anti MDA-5 DM from Asia were clinically amyopathic with cutaneous vasculopathy and RP-ILD, data from different ethnic backgrounds emerged and showed heterogeneous clinical behavior.^7^

ILD is an important clinical feature seen in 60–100% of patients, with a rapidly progressive phenotype reported in over 40% (20–75%) of the cases. ^1^Anti MDA-5 DM has 20-fold higher odds for RPILD development than MDA-5 negative DM, and it is the most critical factor predicting survival.^8^ RP-ILD presents with worsening dyspnea and cough, with radiographic deterioration causing hypoxia. HRCT in RP-ILD shows features of organizing pneumonia, peribronchovascular and peripheral areas of patchy airspace consolidation and GGOs, septal thickening, or traction bronchiectasis.^9^ In our index case, bilateral GGOs with subtle septal thickening in the background and basal consolidation were identified pointing towards diffuse alveolar injury. At the same time, the presence of pneumomediastinum and pneumothorax suggests the complicated course of the disease.

Histopathological data of RP-ILD associated with CADM with positive Anti MDA-5 antibody is limited. We could find less than ten case reports in English literature, including autopsy and surgical lung biopsy. The predominant pathology is DAD, followed by NSIP and organizing pneumonia pattern of injury.^10^

Acute interstitial pneumonitis has a broad differential diagnosis without obvious muscle involvement. Viral pneumonitis, atypical bacterial pneumonia, and hypersensitivity pneumonitis can show similar HRCT findings. In our case, a thorough work-up was done to rule out bacterial, fungal, and mycobacterial pneumonia, with negative results. Viral PCR performed on the antemortem sample showed positivity for the H1N1 virus. The negative PCR and immunohistochemistry result for H1N1 virus in the postmortem sample could be explained by a reduction in the viral load following the initiation of oseltamivir therapy in our patient. COVID-19 infection could mimic clinical and histopathological features of Anti MDA-5 DM; however, antemortem as well as postmortem tests for COVID-19 PCR were consistently negative. The presentation of the index case was like that of acute interstitial pneumonia, which on histology shows features of organizing DAD. The DAD was caused by the rapid progression of RP-ILD combined with acute H1N1 viral pneumonitis. Pneumomediastinum is a life-threatening complication reported in up to 15% of patients with Anti MDA-5 DM and ILD, and is associated with worse survival (mortality of 60% vs 37% in those without pneumomediastinum).^11^ The HRCT chest, in our case, revealed features of pneumomediastinum and left pneumothorax, with air also dissecting into the neck spaces.

Ro-52 antibodies are described in up to 60% of patients with Anti MDA-5 DM and may pose a greater risk for RP ILD and mortality in these patients.^12^ In our case, the association of Ro52 antibody with Anti MDA-5 antibody was an indicator of poor prognosis.

The distinct histopathological features of classical DM, like perifascicular atrophy, perivascular T-lymphocyte-rich inflammation, myonecrosis, myophagocytosis, are uncommon in Anti MDA-5 DM.^2^ In our case, multiple groups of skeletal muscle were evaluated, and extensive work-up did not reveal any significant histological features of DM, barring mild fiber size variation.

There is an increased prevalence of arthritis (42-82%) and arthralgia in patients with Anti MDA-5 DM that closely resembles the features of rheumatoid arthritis, potentially leading to misdiagnosis in the absence of other DM symptoms.^2^

DM treatment is based on the long-term administration of high doses of glucocorticoids. Immune-modulating agents (cyclophosphamide, azathioprine, or mycophenolate mofetil (MMF)) are considered second-line treatment.^13^ Intravenous immunoglobulin and immunomodulators like cyclosporine A (CyA), and Tacrolimus (Tac), are also recommended as add-on therapy.^14^ It is advisable to treat the patients with fulminant disease aggressively.

## CONCLUSION

Anti MDA-5 DM has recently been identified as a subtype of myositis characteristically associated with RP-ILD. The clinical phenotype is highly varied in different ethnicities. The initial symptoms in our patient were non-specific. Later, the RP-ILD of CADM in our patient mimicked acute respiratory distress syndrome caused by viral infection. Hence, the definite diagnosis could not be made during ante-mortem work-up. The role of autopsy, in this case, is immense, as it revealed the underlying interstitial lung disease, and the CADM could be confirmed on postmortem serological investigations. Awareness of the condition and its potential manifestations is the key to early detection and improved survival.
